# FG-4592 protects the intestine from irradiation-induced injury by targeting the TLR4 signaling pathway

**DOI:** 10.1186/s13287-022-02945-6

**Published:** 2022-06-21

**Authors:** Zhenlan Feng, Qinshu Xu, Xiang He, Yuedong Wang, Lan Fang, Jianpeng Zhao, Ying Cheng, Cong Liu, Jicong Du, Jianming Cai

**Affiliations:** 1 School of Public Health and Management, Wenzhou Medical University, Wenzhou, 325000 Zhejiang Province China; 2 Department of Radiation Medicine, Faculty of Naval Medicine, Naval Medical University, Shanghai, 200433 China; 3 College of Basic Medicine, Naval Medical University, Shanghai, 200433 China

**Keywords:** Radiation, Intestine injury, FG-4592, Intestinal stem cell, TLR4

## Abstract

**Background:**

Severe ionizing radiation (IR)-induced intestinal injury associates with high mortality, which is a worldwide problem requiring urgent attention. In recent years, studies have found that the PHD-HIF signaling pathway may play key roles in IR-induced intestinal injury, and we found that FG-4592, the PHD inhibitor, has significant radioprotective effects on IR-induced intestinal injury.

**Methods:**

In the presence or absence of FG-4592 treatment, the survival time, pathology, cell viability, cell apoptosis, and organoids of mice after irradiation were compared, and the mechanism was verified after transcriptome sequencing. The data were analyzed using SPSS ver. 19 software.

**Results:**

Our results show that FG-4592 had significant radioprotective effects on the intestine. FG-4592 improved the survival of irradiated mice, inhibited the radiation damage of intestinal tissue, promoted the regeneration of intestinal crypts after IR and reduced the apoptosis of intestinal crypt cells. Through organoid experiments, it is found that FG-4592 promoted the proliferation and differentiation of intestinal stem cells (ISCs). Moreover, the results of RNA sequencing and Western blot showed that FG-4592 significantly upregulated the TLR4 signaling pathway, and FG-4592 had no radioprotection on TLR4 KO mice, suggesting that FG-4592 may play protective role against IR by targeting TLR4.

**Conclusion:**

Our work proves that FG-4592 may promote the proliferation and regeneration of ISCs through the targeted regulation of the TLR4 signaling pathway and ultimately play radioprotective roles in IR-induced injury. These results enrich the molecular mechanism of FG-4592 in protecting cells from IR-induced injury and provide new methods for the radioprotection of intestine.

## Introduction

Acute radiation exposure is a serious public and military health problem [1]. Ionizing radiation (IR) can cause radiation damage. Intestinal tissues are extremely sensitive to IR, which is a common pathological effect after exposure to high doses of IR [2]. When the radiation dose is ≥ 10.0 Gy, intestinal tissue necrosis can ensue, leading to the death of patients with acute intestinal radiation sickness [3]. In addition, IR-induced intestinal injury is a common complication of abdominal tumors, which limits the dose and efficacy of radiotherapy for patients with tumors [4]. Although great progress has been made in the radioprotection, there is still no ideal treatment for IR-induced intestinal injury caused by high-dose radiation, which is one of the key problems needing urgent attention in the field.

Over the years, there have been many studies on the mechanism and prevention of intestinal ionizing radiation injury [5, 6]. It was found that acute IR-induced intestinal injury is a complex process involving cell death and inflammatory activation [7]. IR can directly damage intestinal cells, leading to the death of intestinal epithelial cells and intestinal crypt stem cells and the destruction of the epithelial barrier, resulting in digestion and absorption disorders, electrolyte imbalances, and even bacterial translocation, which is the main cause of death caused by intestinal radiation injury [8]. In addition, if the intestine is exposed to a large doses of IR, then swelling and apoptosis of microvascular endothelial cells will ensue, causing the release of inflammatory factors, the formation of microthrombosis, and the formation intestinal tissue ischemia and hypoxia, thereby aggravating intestinal injury [9]. Much research has been carried out in the prevention and treatment of IR-induced intestinal injury, including stem cell transplantation, cytokines treatment, drug development, comprehensive therapy, and so on [10–12]. The progress in basic has been made from several different aspects, but the overall radioprotective effects are not ideal [13]. For example, results show that some compounds have no obvious therapeutic effect. Although some compounds can alleviate the occurrence and development of intestinal ionizing radiation injury, these compounds cannot be directly applied in clinical treatment due to their high toxicity or some unpredictable side effects [14].

The PHD-HIF signaling pathway, a research hotspot in recent years, is involved in inflammation, cancer, immunity and other physiological processes [15, 16]. HIF (hypoxia-inducible factor) is a DNA-binding protein consisting of an *α* subunit (HIF-1α or HIF-2α) and a *β* subunit (HIF-1β). Since it was discovered by Semenza et al. in the 1990s, the role of HIF in hypoxia tolerance, tumor proliferation, erythropoiesis and other aspects has been gradually recognized [17]. PHD (HIF prolyl hydroxylase), an important negative regulator of HIF, can reduce the stability of HIF-1α through hydroxylation. The PHD-HIF pathway is not only related to hypoxia, but also involved in various pathophysiological processes such as inflammation, tumor development and immunity [18, 19].

In recent years, it has been found that the PHD-HIF pathway is a new target for the treatment of IR-induced intestinal injury [20]. For example, dimethyloxalylglycine (DMOG), as a PHD inhibitor, has been reported to mediate radioprotection mainly through the inhibition of PHD2 and the upregulation of HIF-2α [21]. Our group has previously reported that FG-4592 has significant radioprotection effects on the hematopoietic system [22]. FG-4592, also known as Roxadustat, is a novel oral PHD inhibitor. It was first used in the treatment of anemia to enhance the synthesis and release of EPO by inhibiting PHD and increasing HIF level [23]. In 2014, FG-4592 was applied to China for clinical trial as a class 1.1 new drug. Now, phase III clinical study has been carried out in global setting, and it is safe for human use [24–26]. In this study, we report that FG-4592 protects the intestine from IR-induced injury by targeting the TLR4 signaling pathway and FG-4592 might be a potentially highly effective and selective intestinal radioprotector.

## Materials and methods

### Chemicals and reagents

FG-4592 was purchased from Cayman Chemical Company (www.caymanchem.com), and normal saline (NS) was obtained from Changhai Hospital (Shanghai, China). The apoptosis detection kit was purchased from TransGen (Beijing, China). The PCR kit (RR036A and RR420A) was purchased from TAKARA (Japan). RPMI 1640, DMEM and fetal bovine serum (FBS) were supplied by Gibco (New York, USA). Organoid cultures were obtained from STEM CELL. BCL2, BAX, C-CASPASE3, GAPDH, NF-κB and P-IKK-β antibodies were supplied by CST. The TLR4 antibody was supplied by Proteintech. In Situ Cell Death Detection Kit was obtained from Roche (Basel, Switzerland). The primes were obtained from Shenggong Biotech (Shanghai, China). The list of primers is shown in Table 1.

**Table 1:** qRT-PCR primers for the eight genes evaluated

Gene symbol	Forward	Reverse
TLR4	AAATGCACTGAGCTTTAGTGGT	TGGCACTCATAATGATGGCAC
IL-6	CTGCAAGAGACTTCCATCCAG	AGTGGTATAGACAGGTCTGTTGG
IGFBP2	CAGACGCTACGCTGCTATCC	CCCTCAGAGTGGTCGTCATCA
SOX2	GCGGAGTGGAAACTTTTGTCC	CGGGAAGCGTGTACTTATCCTT
REG2	CTGATGTTCCTGTCATACAGCC	CCAGGTCAAACGGTCTTCAATTA
REG3B	ACTCCCTGAAGAATATACCCTCC	CGCTATTGAGCACAGATACGAG
REG3D	GACTCCATGATCTGTCACTTGG	CATAGGGAAATGTTGGGTCACAA
HK1	CAAGAAATTACCCGTGGGATTCA	CAATGTTAGCGTCATAGTCCCC

### Cell culture and treatment

MODE-K cells (normal mouse intestinal epithelial cell line) was obtained from American Type Culture Collection and cultured in RPMI 1640 with 10% FBS at 37 °C in a 5% CO_2_ humidified chamber. HIEC cells (CRL-3266™, normal human intestinal epithelial cell line) was obtained from American Type Culture Collection and cultured in DMEM with 10% FBS at 37 °C in a 5% CO_2_ humidified chamber. After reviewing the literature and CCK-8 pre-experiments, cells were treated with FG-4592 (10 μM) for 12 and 2 h before irradiation [27].

### Cell viability and apoptosis assay

Cell viability was analyzed by the CCK-8 kit. Pre-treated cells were seeded into 96-well plates at 5000 cells/well. The cells were counted by absorbance measurements at 450 nm approximately 24 h post-radiation. Apoptosis was analyzed using the apoptosis detection kit. After radiation, the cells were stained using Annexin V-fluorescein isothiocyanate (AV-FITC) and propidium iodide-phycoerythrin (PI-PE). The cells were then analyzed by flow cytometry (Beckman CytoFLEX) in accordance with the manufacturer’s instructions.

### Animals and treatment

Male C57BL/6 mice were obtained from China Academy of Science (Shanghai, China). TLR4 KO mice aged 6–8 weeks old were purchased from the Model Animal Research Center, Nanjing University. All mice were housed in a laboratory animal room under standard conditions. The experiments were approved by the Laboratory Animal Center of the Naval Medical University, China, in conformance with the National Institute of Health Guide for the Care and Use of Laboratory Animals. The mice were treated with FG-4592 (25.0 mg/Kg, dissolved in NS) via peritoneal injection 24 and 2 h before IR.

### Irradiation

^60^Co (Naval Medical University, China) was used to irradiate the mice and cells. The mice were irradiated at 8.0 or 8.5 Gy to establish the total body irradiation (TBI) model and irradiated at 25.0 Gy at establish the abdominal irradiation (ABI) model.

### Histological examination

Small intestinal tissues were removed from mice and then fixed in 4% paraformaldehyde after IR. Hematoxylin and eosin (HE), TUNEL (terminal deoxynucleotidyl transferase dUTP nick-end labeling) staining and Ki67 staining were performed according to the manufacturer’s instructions. The TUNEL^+^ cells were counted in 10 crypts per section. The Ki67 positive area per section was measured using ImageJ software (National Institutes of Health, Bethesda, MD, USA).

### FISH (Fluorescence in situ hybridization)

FISH was used to detect the expression of Lgr5^+^, the intestinal stem cell marker, in intestinal tissues. Small intestinal tissues were removed from mice and then fixed in 4% paraformaldehyde after IR. FISH was conducted according to the manufacturer’s instructions. Fluorescence microscopy was used to observe FISH results.

### Intestinal organoid culture

The small intestine was removed from the mesentery and rinsed with cold PBS after longitudinal incision. The villi were gently scraped, and the remaining tissue was washed approximately 10 times with cold PBS. The tissue was cut into 2–3 mm fragments, transferred to 15 mM EDTA/PBS without Ca^2+^/Mg^2+^, and incubated on ice at 4 °C for 1 h. After incubation, the tissue fragments were shaken vigorously and pelleted approximately three times with cold PBS at 290 rpm for 5 min. Approximately 200 isolated crypts were embedded in Matrigel (Corning, New York, USA) and cultured in crypt culture medium (IntestiCult™ Organoid Growth Medium, STEM CELL, Canada) [28–31]. The intestinal organoid culture medium was changed every 3 days. The mature organoids were observed under a microscope, and the surface area and budding situation of the organoids were measured using ImageJ software. The TUNEL detection kit was used to detect the apoptotic ability of the irradiated organoid.

### Intestine immunofluorescence

Immunofluorescence analysis was used to detect OLFM4. The intestinal tissues or intestinal organoid was fixed in 4% paraformaldehyde for 20 min and permeabilized in 0.5% Triton X-100 for 10 min. After blocking in BSA, the intestinal tissues or intestinal organoid was stained with antibodies, followed by the secondary antibody (1:1000). The images were obtained with a fluorescent microscope.

### RNA sequencing and functional enrichment analysis

Total RNA was isolated from the intestine of mice using Trizol (Invitrogen, USA) at 24 h after radiation. NanoVue (GE, USA) was used to assess RNA purity. Each RNA sample had an A260/A280 ratio greater than 1.8 and an A260/A230 ratio greater than 2.0. Sequencing was performed at Oebiotech (Shanghai, China) with the Illumina HiSeq X Ten. Prior to sequencing, the raw data were filtered to produce high-quality clean data. All the subsequent analyses were performed using the clean data.

### Statistical analysis

Data were expressed as means ± the standard errors of means. Two-tailed Student’s t test was used to analyze the differences between two groups. One-way ANOVA was employed to analyze the differences among three groups. Kaplan–Meier analysis was applied to estimate the difference of overall survival between two groups. The data were analyzed using SPSS ver. 19 software (IBM Corp, Armonk, New York, USA). *P* < 0.05 was considered statistically significant.

## Results

### FG-4592 exhibited a significant radioprotective effect in vivo and in vitro

**Fig. 1:**
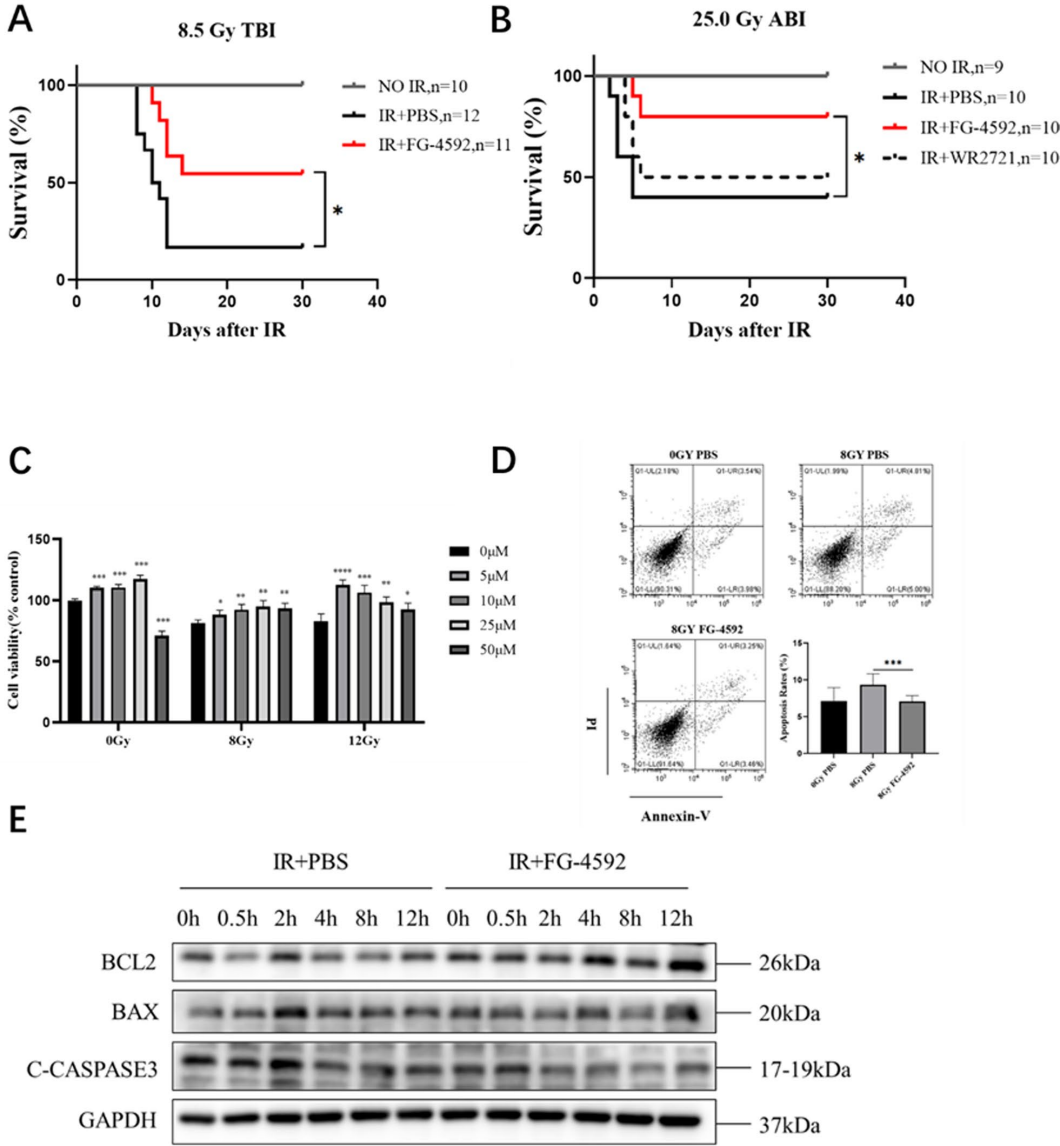
FG-4592 exhibited a significant radioprotective effect in vivo and in vitro. **A** The survival of mice after total body irradiation of 8.5 Gy. **B** The survival of mice after local abdominal irradiation of 25.0 Gy. **C** Effects of different concentrations of FG-4592 on MODE-K cell viability was measured by the CCK-8 assay. **D** HIEC cell apoptosis was detected by flow cytometry. **E** Expression of HIEC cell apoptosis pathway-related proteins. The data were presented as mean ± SD. *P < 0.05, **P < 0.01, and ***P < 0.001 for control versus FG-4592 treatment

To prove the radioprotective effects of FG-4592 on IR-induced intestinal injury, we used C57BL/6 mice as the research object. C57BL/6 mice were given 25.0 mg/kg FG-4592 intraperitoneally 24 and 2 h before irradiation, followed by total body irradiation of 8.5 Gy or local abdominal irradiation of 25.0 Gy. FG-4592 could significantly improve the survival rate of mice after IR (Fig. 1A, B). Meanwhile, MODE-K cells and HIEC cells were treated with FG-4592 at 10 μM 12  and 2 h before irradiation; cell viability and apoptosis rate were detected 24 h after irradiation. Compared with the IR group, the cell viability after irradiation was significantly increased and the apoptosis rate was decreased in the FG-4592 treatment group (Fig. 1C, D). Meanwhile, the levels of apoptosis-related proteins were detected by using Western blot (WB). FG-4592 reduced the levels of BAX and cleaved CASPASE-3, two apoptosis-promoting proteins, which were up‐regulated by IR and increased BCL2 but inhibited apoptosis and were down‐regulated after IR (Fig. 1E).

### FG-4592 protected the intestinal tissue against radiation-induced injury

**Fig. 2:**
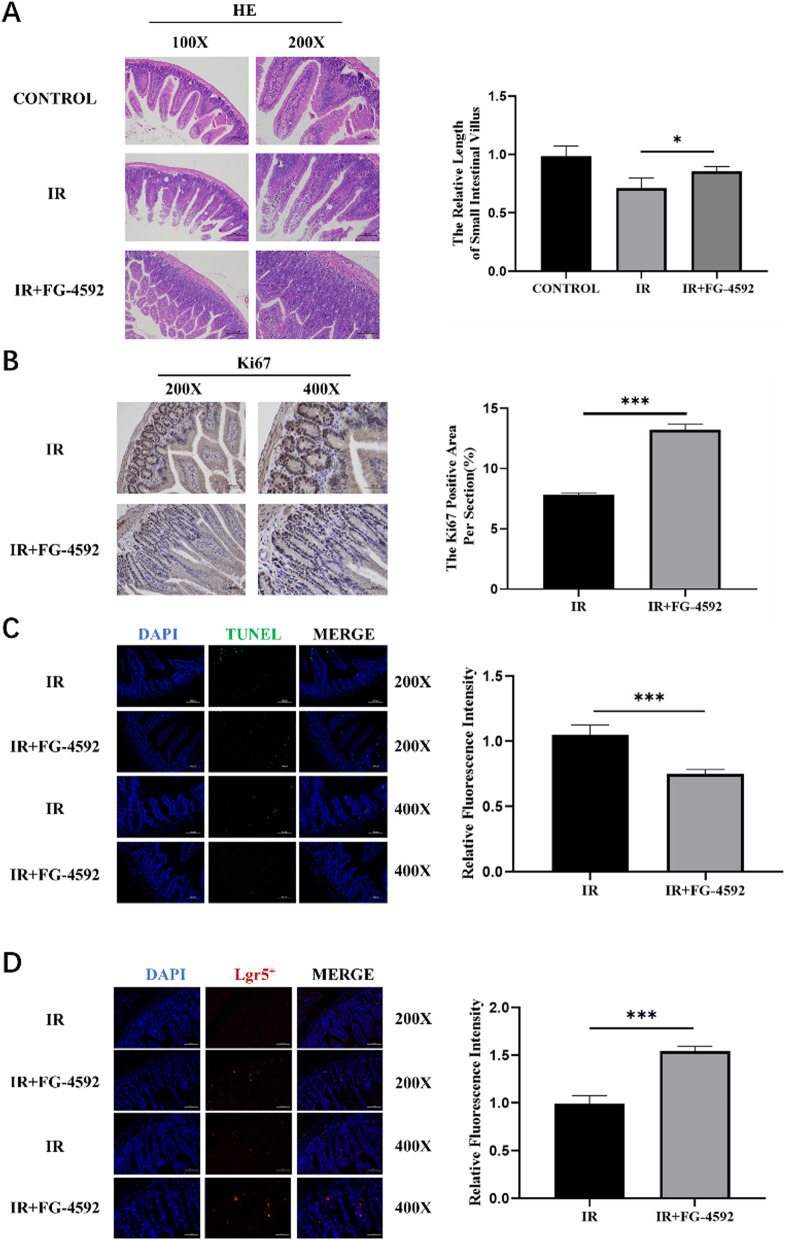
FG-4592 protected the intestinal tissue against radiation-induced injury. C57BL/6 mice were pre-treated with PBS or FG-4592 before 8.5 Gy TBI. **A** Representative images of HE-stained intestinal sections with the indicated treatment at 3.5 d after TBI. **B** The representative images of Ki67-stained intestinal sections with the indicated treatment at 3.5 d after TBI. **C** The representative images of TUNEL-stained intestinal sections with the indicated treatment at 3.5 d after TBI. **D** The representative images of Lgr5^+^ FISH intestinal sections with the indicated treatment at 3.5 d after TBI. The data were presented as mean ± SD. *P < 0.05, **P < 0.01 and ***P < 0.001 for control versus FG-4592 treatment

Subsequently, intestinal tissues of mice were collected 3.5 days after IR for HE staining to observe the degree of intestinal injury (Fig. 2A). HE staining results showed that FG-4592 could improve the intestinal integrity of mice after irradiation, and the villi length and crypt cell number were better than that of the IR group. Ki67 staining showed that the FG-4592 group exhibited greater intestinal structure, taller villi, and more surviving crypts than the control group (Fig. 2B). TUNEL staining showed that FG-4592 could significantly inhibit the apoptosis of intestinal crypts after radiation injury (Fig. 2C). Lgr5^+^ FISH also showed that irradiation could significantly reduce the number of Lgr5^+^ ISCs, while FG-4592 significantly increased the number of Lgr5^+^ ISCs (Fig. 2D). These results proved that FG-4592 had great radioprotective effects on IR-induced intestinal injury.

### FG-4592 protected the intestinal organoid against radiation-induced injury

**Fig. 3:**
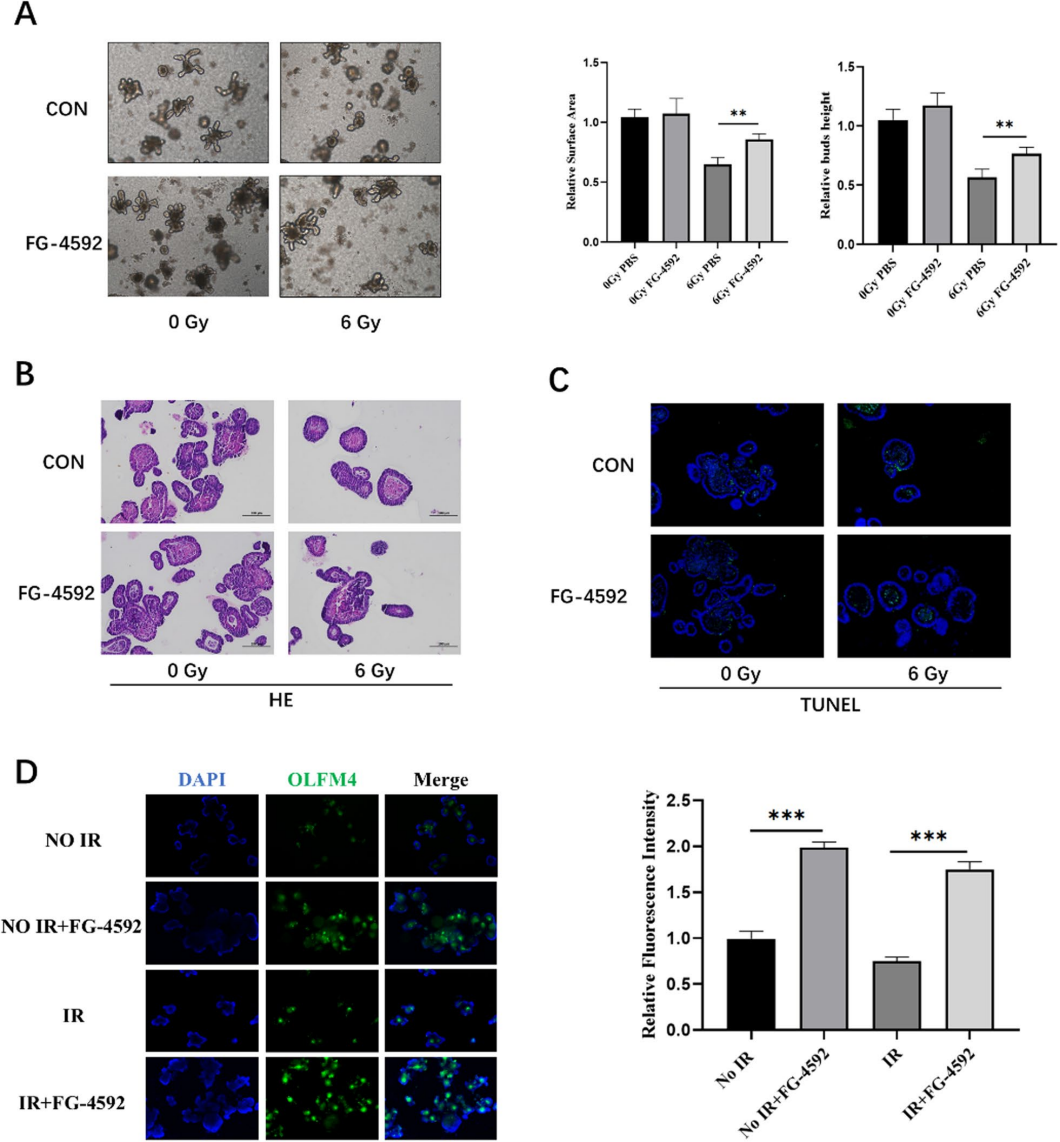
FG-4592 protected the intestinal organoid against radiation-induced injury. **A** Organoid regeneration after IR with PBS and FG-4592 treatment. **B** Representative images of HE-stained intestinal organoids with the indicated treatment after IR. **C** Representative images of TUNEL-stained intestinal organoids with the indicated treatment after IR. **D** The expression of the OLFM4 receptor was detected by fluorescence microscopy. The data were presented as mean ± SD. *P < 0.05, **P < 0.01, and ***P < 0.001 for control versus FG-4592 treatment

The intestinal organoid is a great technology to study the proliferation, differentiation, and regeneration of ISCs [32]. In this work, intestinal organoids were also used to explore the radioprotective effects of FG-4592 on intestinal radiation injury. Intestinal crypts of C57BL/6 mice were extracted for organoid culture, and then it was stimulated with FG-4592 (50 μM) before 6.0 Gy IR. As shown in Fig. 3A, FG-4592 could improve the ability of organoid formation. Compared with the IR group, the number and volume of single organoid bud in the FG-4592 group were increased. At the same time, HE and TUNEL staining were also performed on the organoids, and it was found that FG-4592 could significantly promote the proliferation and inhibit apoptosis of the intestinal organoids after 6.0 Gy (Fig. 3B, C). These results were consistent with the results of intestinal tissues in mice. OLFM4 is another ISCs marker, and immunofluorescence analysis was used to detect the expression of OLFM4 in intestinal organoid (Fig. 3D). FG-4592 increased the expression of OLFM4, which was down‐regulated by IR. These results suggested that FG-4592 could significantly improve the proliferation and differentiation of irradiated ISCs.

### Identification of DEGs after FG-4592 treatment

**Fig. 4:**
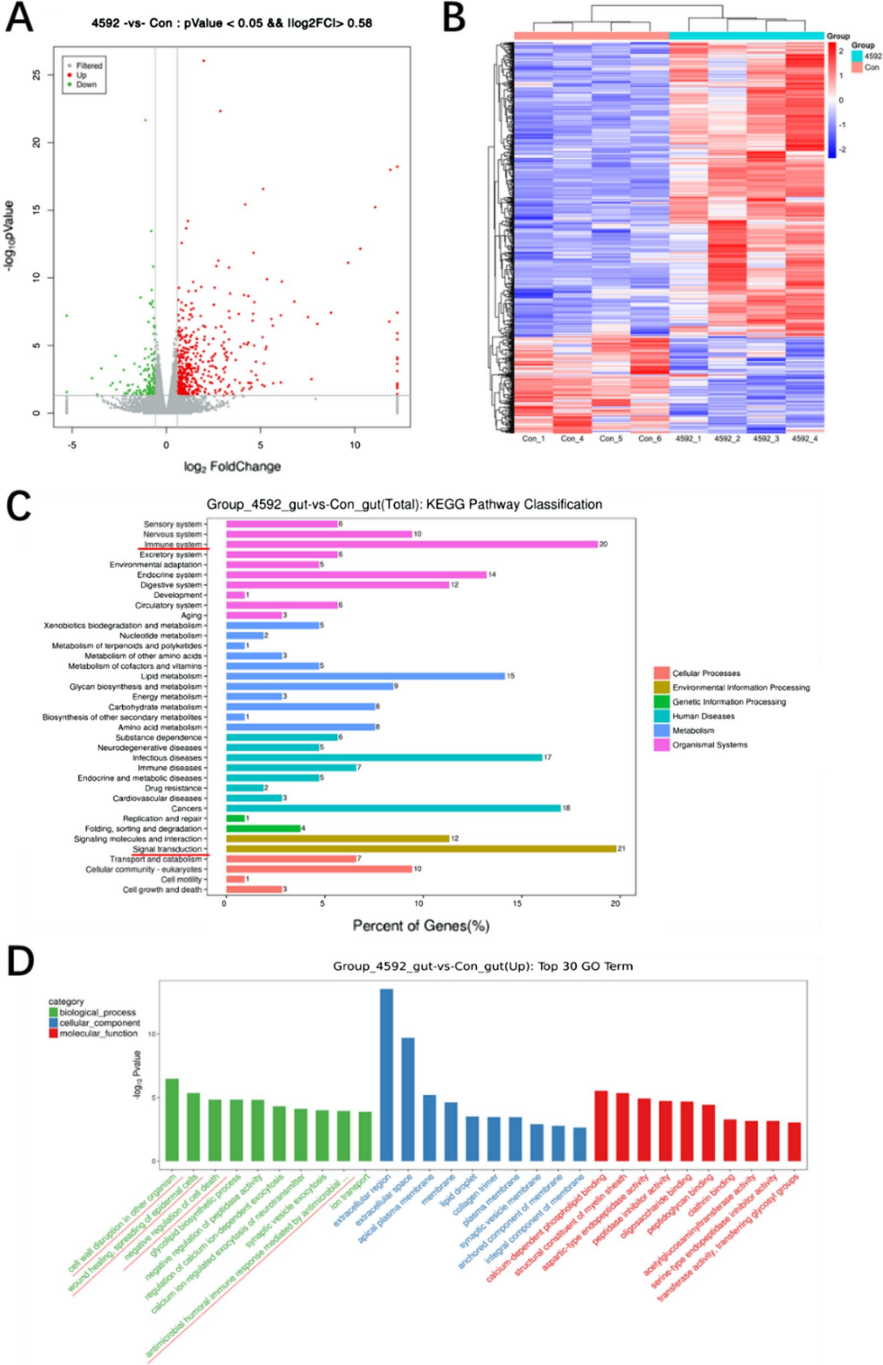
Identification of DEGs after FG-4592 treatment. **A** Scatter plot of differently expressed genes in gut tissue after FG-4592 treatment. Each dot stands for a gene. Red and green color dots indicate an increase or decrease, respectively. **B** Heat map of differential genes expression between wild-type mice and FG-4592 mice. **C** Pathway enrichment analysis of KEGG pathways within the core network. **D** GO term analysis was performed on differentially expressed genes

To explore the regulatory mechanism of FG-4592 on intestinal radiation injury, RNA sequencing (4:4) of intestinal tissues was conducted. A total of 476 up-regulated and 157 down-regulated differentially expressed genes (DEGs) were identified (Fig. 4A). The differential gene heat map was drawn for analysis (Fig. 4B). At the same time, KEGG and GO analyses were conducted to identify the significant enrichment pathways of DEGs (Fig. 4C, D). The KEGG results showed that immune system and signal transduction were significantly enriched (Fig. 4C), while the GO results showed that cell wall disruption in other organisms (GO:0044278), wound healing, spreading of epidermal cells (GO:0035313), negative regulation of cell death (GO:0060548) and antimicrobial humoral immune response mediated by antimicrobial peptide (GO:0061844) were significantly enriched (Fig. 4D and Table 2). These pathways have been reported to be related to the process of IR-induced injury [33, 34].

### TLR4 signaling pathway plays a critical role in the radioprotection of FG-4592

**Fig. 5:**
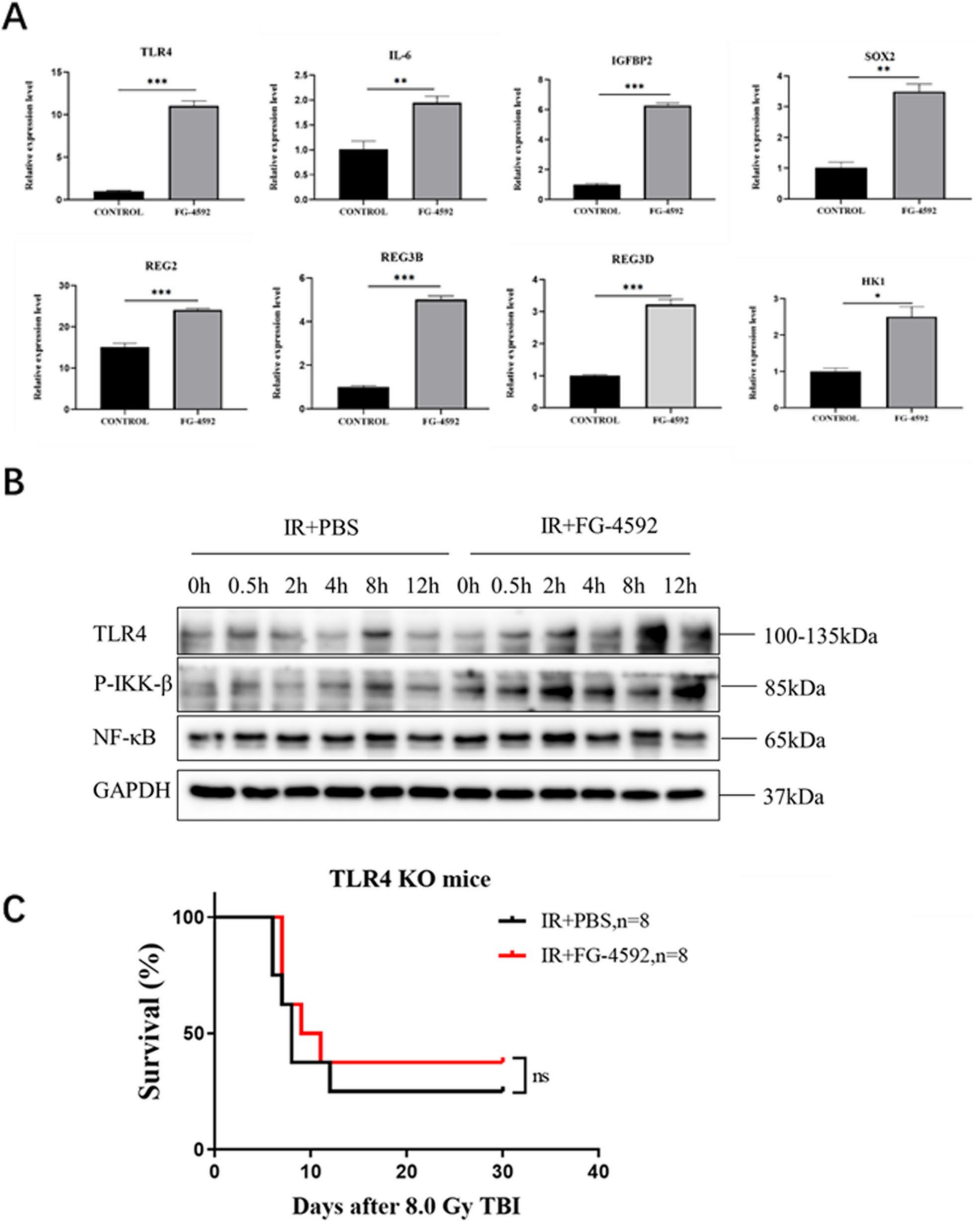
FG-4592 activates TLR4-related pathways. **A** RNA level to verify the expression of DEGs, including IL-6, IGFBP2, SOX2, HK1, REG3B, REG2 and REG3D. **B** The expression of TLR4 pathway-related proteins. **C** The radioprotection of FG-4592 on TLR4 KO mice

In the list of DEGs (Table 3), we selected TLR4 as a key downstream molecule. In our previous works, TLR4 played a key role in the process of IR-induced injury. Through quantitative PCR, we found that FG-4592 could significantly upregulate the mRNA level of TLR4. IL-6, IGFBP2, SOX2, HK1, REG3B, REG2, REG3D and other DEGs were significantly upregulated (Fig. 5A). Moreover, the changes in the TLR4 signaling pathway of FG-4592 treated MODE-K cells were also increased by using WB. The results of WB showed that the expression of TLR4 and Phospho-IKK-β was increased after FG-4592 treatment (Fig. 5B). Next, TLR4 KO mice were used to verify the function of TLR4 in FG-4592-induced radioprotection. TLR4 KO mice were divided into the IR + PBS and the IR + FG-4592 groups. After recording the survival period after 8.0 Gy IR, we found that the mortality rate of TLR4 KO mice was not statistically significant between the IR + PBS group and the IR + FG-4592 group (Fig. 5C), indicating that TLR4 KO reversed the radioprotection of FG-4592. These results proved that the radioprotection of FG-4592 was caused by TLR4.

**Table 2:** List of top 30 GO terms

ID	Term	Category	P val	Enrichment_score
**GO:0044278**	**Cell wall disruption in other organism**	**Biological_process**	**0.000**	**33.89672131**
**GO:0035313**	**Wound healing, spreading of epidermal cells**	**Biological_process**	**0.000**	**20.33803279**
GO:0009247	Glycolipid biosynthetic process	Biological_process	0.000	15.64464061
**GO:0060548**	**Negative regulation of cell death**	**Biological_process**	**0.000**	**5.332068521**
GO:0010466	Negative regulation of peptidase activity	Biological_process	0.000	4.635449068
GO:0006811	Ion transport	Biological_process	0.000	2.433610761
GO:0017158	Regulation of calcium ion-dependent exocytosis	Biological_process	0.000	8.217386985
GO:0048791	Calcium ion-regulated exocytosis of neurotransmitter	Biological_process	0.000	7.532604736
GO:0016079	Synaptic vesicle exocytosis	Biological_process	0.000	7.136151855
**GO:0061844**	**Antimicrobial humoral immune response mediated by antimicrobial peptide**	**Biological_process**	**0.000**	**5.467213115**
GO:0005576	Extracellular region	Cellular_component	0.000	2.479256595
GO:0005615	Extracellular space	Cellular_component	0.000	2.321368755
GO:0016020	Membrane	Cellular_component	0.000	1.440847291
GO:0016324	Apical plasma membrane	Cellular_component	0.000	3.122066437
GO:0016021	Integral component of membrane	Cellular_Component	0.000	1.367007432
GO:0009897	External side of plasma membrane	Cellular_component	0.000	2.965963115
GO:0005886	Plasma membrane	Cellular_component	0.000	1.421755063
GO:0009986	Cell surface	Cellular_component	0.001	2.135226539
GO:0005811	Lipid droplet	Cellular_component	0.001	4.580638015
GO:0005581	Collagen trimer	Cellular_component	0.001	4.519562842
GO:0019911	Structural constituent of myelin sheath	Molecular_function	0.000	20.33803279
GO:0005544	Calcium-dependent phospholipid binding	Molecular_function	0.000	7.975699132
GO:0004190	Aspartic-type endopeptidase activity	Molecular_function	0.000	10.84695082
GO:0070492	Oligosaccharide binding	Molecular_function	0.000	14.52716628
GO:0030414	Peptidase inhibitor activity	Molecular_function	0.000	4.519562842
GO:0042834	Peptidoglycan binding	Molecular_function	0.000	12.71127049
GO:0004198	Calcium-dependent cysteine-type endopeptidase activity	Molecular_function	0.000	10.70422778
GO:0030246	Carbohydrate binding	Molecular_function	0.001	3.097162353
GO:0030276	Clathrin binding	Molecular_function	0.001	5.021736491
GO:0008375	Acetylglucosaminyltransferase activity	Molecular_function	0.001	6.163040238

The [bold] signaling pathways and DEGs have been shown that they can play radioprotective roles in the process of IR induced intestinal injury

**Table 3:** Partial differential genes list

Gene_id	FoldChange	*P* val	Regulation
**Hk1**	**1.46035**	**0.001071**	**Up**
**IL-6**	**2.013457**	**0.08727**	**Up**
**Tlr4**	**2.277509**	**1.70E-05**	**Up**
**Igfbp2**	**2.345283**	**0.023405**	**Up**
**Reg3b**	**2.837572**	**0.008005**	**Up**
**Reg3d**	**3.050849**	**0.006954**	**Up**
**Sox2**	**6.727548**	**0.000878**	**Up**
**Reg2**	**17.74323**	**0.004824**	**Up**
Pde2a	1.633734	0.002347	Up
Pglyrp1	1.698371	0.001095	Up
Arhgap24	1.914116	9.08E-05	Up
Nov	2.23212	0.004262	Up
Defa28	2.509691	0.018393	Up
Agr2	2.671268	1.54E-06	Up
Clu	3.250905	8.03E-06	Up
Wnt11	3.569708	0.000169	Up
Plet1	3.675288	1.35E-05	Up
Ltf	3.68403	0.000989	Up
Scnn1g	5.190979	0.000447	Up
Ntf5	5.831077	0.003121	Up
Scnn1b	7.607157	6.84E-06	Up

The [bold] signaling pathways and DEGs have been shown that they can play radioprotective roles in the process of IR induced intestinal injury

## Discussion

Acute severe radiation-induced damage is a worldwide problem [1]. Although researchers have made some progress in this field, there is still no effective means for the prevention and treatment of acute intestinal radiation sickness caused by high-dose irradiation [35]. Therefore, it is of great significance to figure out the key mechanism of IR-induced injury and to identify new radioprotective agents.

In recent years, it has been reported that the PHD-HIF pathway is a new target for the treatment of treated IR-induced intestinal injury [20]. Taniguchi CM et al. reported that a radioprotective compound DMOG can regulate the PHD2-HIF pathway. At 16.0 Gy lethal TBI or 20.0 Gy ABI in mice, the survival rate of the DMOG-treated mice was significantly improved, with 2/3 of the mice still alive 60 days after IR exposure, and all untreated control animals died 10 days after IR. The authors also found that DMOG-mediated radioprotection was achieved mainly through inhibition of PHD2 and upregulation of HIF-2 [21]. Forristal CE et al. reported similar results, that is, DMOG played radioprotective effects on the hematopoietic system through the PHD-HIF pathway [36]. Cummins EP et al. demonstrated that the upregulation of HIF with a PHD inhibitors could promote NF-κB translocation by activating IKK-β [37], while Greten FR et al. demonstrated that the activation of NF-κB in intestinal epithelial cells could inhibit their apoptosis [38]. Olcina and Giaccia published a review in *J Clin Invest* suggesting that the upregulation of the HIF pathway, especially the inhibition of PHD, may be a new and effective way for the prevention and treatment of IR-induced intestinal injury [20].

In this study, we systematically studied the radioprotective effects of PHD inhibitor FG-4592 on IR-induced intestinal injury and found that FG-4592 significantly improved the survival of mice exposed to TBI and ABI. Moreover, FG-4592 also promoted cell proliferation and inhibited cell apoptosis. Furthermore, HE staining, Ki67 staining, and TUNEL staining of intestinal tissue showed that FG-4592 could significantly reduce the damage degree of intestinal tissue, promote the proliferation of intestinal crypt cells and reduce the apoptosis of intestinal crypt cells caused by IR. Lgr5^+^ FISH also showed that FG-4592 significantly increased the number of Lgr5^+^ ISCs.

Intestinal organoids have unlimited proliferation ability and can simulate many characteristics of the intestine, which is an important model for studying intestinal development, function and diseases. In 2009, Sato et al. successfully cultured single ISCs into a three-dimensional structure containing crypt-like regions and villous epithelial region that could grow and differentiate into all types of intestinal epithelial terminal cells, which can accurately simulate the physiological state of intestinal epithelium and provide a new research approach for ISCs [30]. In this study, we constructed an intestinal organoid culture system and found that FG-4592 could improve the ability of crypt organoid formation. Compared with the IR group, the FG-4592 group had more extensive crypt organoid formation, and the number and volume of single organoid bud were increased. HE staining and TUNEL staining of intestinal organoids showed that FG-4592 could promote the proliferation of intestinal organoids and inhibit the apoptosis of intestinal organoids after IR. In addition, the OLFM4 immunofluorescence assay confirmed that FG-4592 significantly upregulated the number of ISCs in intestinal organoids after IR.

As a high-throughput sequencing method, RNA sequencing technology is used to study all mRNAs produced by a specific organ or cell in a state of characteristic function. In this study, RNA sequencing was used to further investigate the potential mechanism of FG-4592, and 633 DEGs were identified, including 476 up-regulated DEGs and 157 down-regulated DEGs, using |log2 Fold Change|> 0.58 and *P* < 0.05 as the criteria. Subsequently, we conducted heat map analysis, KEGG analysis and GO analysis for DEGs between the two groups and found that cell wall disruption in other organism (GO:0044278), wound healing, spreading of epidermal cells (GO:0035313), negative regulation of cell death (GO:0060548) and antimicrobial humoral immune response mediated by antimicrobial peptide (GO:0061844) were significantly enriched. These pathways have been reported to the regulation of radiation damage. In the expression verification of DEGs, we found that FG-4592 could significantly upregulate the TLR4 mRNA level, and IL-6, IGFBP2, SOX2, HK1, REG3B, REG2, REG3D and other differential genes were significantly upregulated by QT-PCR. TLR4 plays a key role in radioprotection. Next, the changes in the TLR4 signaling pathway after FG-4592 treatment were also increased by using WB, and the WB results showed that FG-4592 could significantly upregulate the TLR4 signaling pathway. Furthermore, TLR4 KO mice were used to verify the function of TLR4 in FG-4592-induced radioprotection, and we found that TLR4 KO reversed the radioprotection of FG-4592. These results proved that the radioprotection of FG-4592 was dependent on TLR4.

## Conclusion

In conclusion, in this study, we explored the radioprotection and mechanism of FG-4592 in IR-induced intestinal injury through animal survival, intestinal organoids, RNA sequencing, and TLR4 KO mice. These results suggest that FG-4592 may protect the intestine from IR-induced injury by targeting the TLR4 signaling pathway.

## Data Availability

All data generated or analyzed during this study are included in this published article and its supplementary information files. RNA sequencing has been uploaded to the GSA database. The assigned accession of the submission is: CRA006921. It can be accessed from the following link: https://bigd.big.ac.cn/gsa/browse/CRA006921.

## Abbreviations

IR: Ionizing radiation
WB: Western blot
ISCS: Intestinal stem cells
HIF: Hypoxia-inducible factor
DMOG: Dimethyloxalylglycine
FBS: Fetal bovine serum
AV-FITC: Annexin V-fluorescein isothiocyanate
PI-PE: Propidium iodide-phycoerythrin
HE: Hematoxylin and eosin
TUNEL: Terminal deoxynucleotidyl transferase dUTP nick-end labeling
DEGs: Differentially expressed genes
ANOVA: Analysis of variance
TBI: Total body irradiation
ABI: Abdominal irradiation
FISH: Fluorescence in situ hybridization
SD: Standard deviation
KEGG: Kyoto Encyclopedia of Genes and Genomes
